# Case report: Ipilimumab and nivolumab in metastatic adrenocortical cancer with high tumor mutational burden

**DOI:** 10.3389/fonc.2024.1406616

**Published:** 2024-06-10

**Authors:** Rebekka Mispelbaum, Tessa Hattenhauer, Franz-Georg Bauernfeind, Jan-Frederic Lau, Peter Brossart, Annkristin Heine

**Affiliations:** ^1^Department of Oncology, Hematology, Immune-Oncology and Rheumatology, University Hospital Bonn, Bonn, Germany; ^2^Institute of Pathology, University Hospital Bonn (UKB), Bonn, Germany

**Keywords:** adrenocortical cancer, immunotherapy, ipilimumab, nivolumab, high mutational burden

## Abstract

In the setting of metastatic adrenocortical cancer, there are limited therapy options such as mitotane and platinum-based chemotherapy with only low response rates. Ipilimumab and nivolumab are approved for several solid cancer types. Tumor mutational burden is one established marker to predict treatment success of immunotherapy and has been associated with improved response rates to immune checkpoint inhibitors. We here present the case of a 68-year-old woman with metastatic adrenocortical cancer and high tumor mutational burden treated with ipilimumab and nivolumab in a fourth-line setting. She showed a stable disease for at least 48 weeks, which is significantly longer than the treatment response to mitotane or platinum-based chemotherapy. To the best of our knowledge, this is the first successful use of a long-term two-drug immunotherapy (48 weeks) in a patient with metastatic adrenocortical cancer and high mutational burden. Ipilimumab and nivolumab should be considered as a new therapy option in this patient group.

## Introduction

Adrenocortical cancer is an aggressive cancer with rapid tumor progression (1). The 5-year survival rate of metastatic disease is less than 15% (2). No promising evidence-based systemic therapy options exist. Neither new targeting agents (in particular linsitinib, an inhibitor of the insulin-like growth factor 1 receptor) nor the combination of various chemotherapies could optimize the overall survival rates in the last years (3). Even for the most established chemotherapy combination consisting of EDP (etoposide, doxorubicin, and cisplatin) and mitotane, in the randomized control study FIRM-ACT, the reported therapy response rate was only 23.2%, median progression-free survival (PFS) was 5.6 months, and median overall survival was 14.8 months (4). Chemotherapy was accompanied by high toxicity and high rates of serious adverse events (58.1%), especially bone marrow toxicity and infections (4). Therefore, there is a huge need for new therapy options for patients with adrenocortical cancer in advanced tumor stages (3). In the last two decades, a better understanding of the interaction of immune cells and tumor cells has arisen (5). Malignant tumors often inhibit the natural immune responses via signal pathways of programmed death–ligand 1 (PD-L1), programmed cell death protein 1 (PD-1), or cytotoxic T-lymphocyte–associated protein 4 (CTLA-4). An inhibition of these molecules, exploiting immune checkpoint inhibitors (CPIs) such as the PD-1 inhibitor nivolumab and the CTLA-4 inhibitor ipilimumab, causes an enhancement of tumor directed CD 8^+^ T cells, which can result in durable remission of different tumor types (6). One discussed predictor for response to immunotherapy is tumor mutational burden (TMB), causing a high rate of neoantigens and thereby enhancing tumor immunogenicity (7). More than 10 mutations per megabase (Mb) are considered as a high TMB status (6). In many tumor types, high TMB revealed as a response marker to CPIs (6). Ipilimumab and nivolumab are approved for different cancer types, for example, for renal cell cancer or malignant melanoma. For adrenocortical cancer, no immune therapy is approved so far. This case report describes the successful use of ipilimumab and nivolumab in a woman with metastatic adrenocortical cancer and high TMB. To the best of our knowledge, this is the first case report evaluating the role of a long-term two-drug immunotherapy (48 weeks) in a patient with metastatic adrenocortical cancer and high TMB.

## Case description

**Figure 1:**
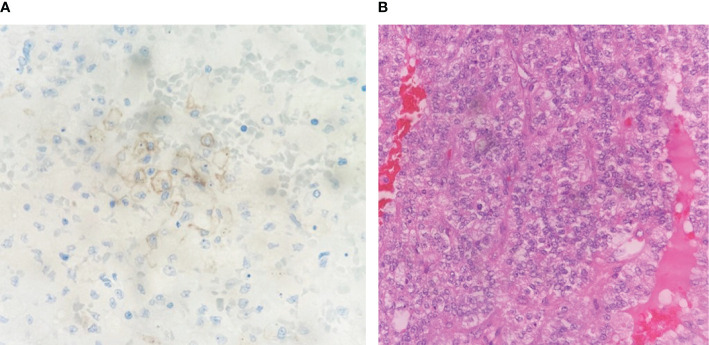
Histology images. **(A)** Essentially solid, suggestive nest-shaped proliferation of moderately atypical cells with partially cleared cytoplasm and blastic nuclei with repeatedly prominent nucleoli. A mitotic figure can be seen slightly to the left below the center of the image (HE, 40×). **(B)** Only very few tumor cells show weak incomplete to complete membranous staining (below 1% of tumor cells). No significant staining of tumor-associated inflammatory cells. (IC 0%). PD-L1 score: TPS <1%; IC 0%; CPS <1. CPS, Combined Positive Score; HE, Hematoxylin and eosin; IC, Immune Cell Score; TPS, Tumor Proportion Score.

**Figure 2:**
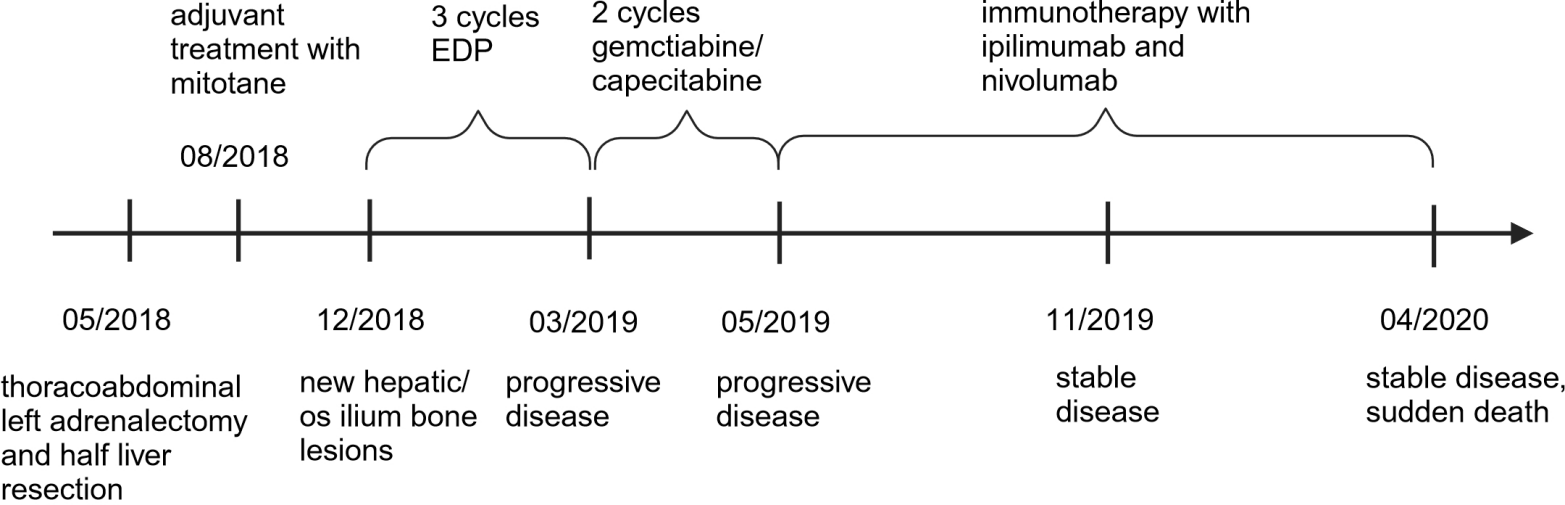
Timeline with relevant data from the episode of care.

A 68-year-old woman presented with a left asymptomatic suprarenal mass on April 2018, detected at sonography routine checkup by her general practitioner. In the physical examination, a resistance was palpable under the left rib. The laboratory tests showed no clinically significant abnormalities. The urine analysis excluded an increased excretion of catecholamines. A CT/MRT scan showed a left suprarenal tumor (11 cm * 9.9 cm * 9.6 cm) with local bordering satellite metastasis and a suspect mass in the liver segment V (1.2 cm). Histology revealed the diagnosis of a PD-L1–negative, hormone-inactive adrenocortical tumor [Tumor Proportion Score (TPS) <1%; Immune Cell Score (IC) 0%; Combined Positive Score (CPS) <1] with one hepatic metastasis (**Figures 1A, B**). A thoracoabdominal left adrenalectomy with a left paraaortic lymphadenectomy and a half-liver resection was performed. The final pathologic examination determined an ENAST Stadium IV with a TNM-stadium pT2, pN1 (2/2) M1, V1, L0, Pn0, and R0. A Lynch syndrome was excluded. In August 2018, an adjuvant treatment with mitotane (joined by hydrocortisone substitution) was started (**Figure 2**). After 4 months, a CT scan showed new hepatic and os ilium bone lesions. Three cycles of EDP, while continuing mitotane therapy, were applied from January until March 2019. Disease progression was diagnosed in March 2019 as a CT scan detected a new lung metastasis and progression of the hepatic and pelvic bone metastasis. The latter was treated with local radiotherapy (30 Gy) because of bone pain. A third-line therapy with two circles of gemcitabine and capecitabine in addition to mitotane therapy was applied from March to April 2019. Chemotherapy and mitotane therapy were interrupted because of tumor progression in May 2019. Based on a high-tissue TMB (10.09 mutations/Mb), identified by the FoundationOne CDx–based Clinical Trial Assay, the patient was included in the study CheckMate 848 (a randomized, open-label, phase 2 study; NCT03668119) and was treated with ipilimumab (1 mg/kg bodyweight, every 6 weeks) and nivolumab (240 mg, every 2 weeks) from May 2019 until death. The interim CT scans in August 2019 (week 12), November 2019 (week 24), and January 2020 (week 36) showed stable disease without new tumor lesions. The patient reported no therapy side effects and a good quality of life. The last interim staging in April 2020 (week 48) confirmed a stable disease. At the end of April, the patient was in hospital treatment because of sickness, emesis, and diarrhea. The symptoms were judged to be an acute event unrelated to the immunotherapy with nivolumab and ipilimumab. In the morning, the patient was found dead most likely because of asphyxiation due to aspiration. An autopsy was not performed.

## Discussion/conclusion

This case report described the successful use of a long-term two-drug immunotherapy in a patient with metastatic adrenocortical cancer and high mutational burden. The initial clinical course of our patient is typical for metastatic adrenocortical cancer and poor prognostic markers, e.g., ENAST-stadium IV. Disease progression during three different chemotherapy regimens (mitotane, EDP, and gemcitabine and capecitabine) corresponds to reported low therapy response rates in several studies and the real-world setting (4, 5).

Combination immunotherapy with ipilimumab and nivolumab showed an excellent durable antitumor activity in our patient, without adverse effects reported. In the literature, the use of PD-1/PD-L1 inhibitor monotherapy in advanced adrenocortical cancer achieved only moderate and heterogeneous therapeutic success, without clear diagnostic markers such as PD-L1, microsatellite instability (MSI), or TMB to select patients with response to immunotherapy (5, 6). There exist only few retrospective and phase I-II studies about CPI treatment with avelumab, pembrolizumab, or nivolumab in this patient group (2, 3, 8, 9). The reported median PFS in these studies was 1.6 to 2.8 months (2, 3, 9). At time of death, our patient had PFS of at least 48 weeks on nivolumab and ipilimumab, without any sign of tumor progression. This favorable tumor control may be explained by combined checkpoint blockade overcoming the known impaired immune activity in adrenocortical cancer (5). For other tumor types such as melanoma and renal cell carcinoma, the phenomenon of superior response to combination immunotherapy is known (10). So far, only one phase II study examined in detail the use of a double CPI treatment (nivolumab and ipilimumab) in advanced adrenocortical cancer in regards to TMB status (10). The subgroup analysis consisted of six patients with metastatic adrenocortical cancer. All patients showing disease control (n = 4; partial response or stable disease) had to stop therapy because of immune-related adverse events, mostly during immunotherapy induction (≤12 weeks). However, in line with our patient, out of these, two subjects had a durable response of 10 and 25 months, despite terminated therapy, thereby emphasizing the therapeutic potential of a two-drug immunotherapy. Interestingly, the two patients with progressive disease at first re-staging in week 12 had an extremely low TMB (10). Our patient had a TMB status of ≥10 mutations/Mb. Many tumor types have shown a positive association between therapy response and high TMB (6, 7). In adrenocortical cancer, TMB may predict therapeutic success for combined checkpoint blockade, as well. While this case represents a single-center report focusing on one patient, additional research is warranted to explore genetic factors and the influence of the tumor microenvironment in relation to immunotherapy within this specific patient cohort.

In summary, our findings may help to support the role of the two-drug immunotherapy ipilimumab and nivolumab in selected patients with advanced adrenocortical cancer with high TMB. Further studies are needed to define the role of TMB (and especially the cut off level) as a predictable marker for immunotherapy success in adrenocortical cancer without neglecting other histological and clinical features (e.g., cortisol production and accompanying mitotane therapy). The ongoing phase II study (NCT02834013) examining nivolumab and ipilimumab in patients with rare cancers might bring new information to this topic in the future.

## Data availability statement

The original contributions presented in the study are included in the article/supplementary material. Further inquiries can be directed to the corresponding author.

## Ethics statement

Written informed consent was obtained from the patient's next of kin for the publication of this case report.

## Author contributions

RM: Conceptualization, Formal Analysis, Investigation, Methodology, Visualization, Writing – original draft, Writing – review & editing. TH: Writing – original draft, Writing – review & editing. F-GB: Writing – review & editing. J-FL: Visualization, Writing – review & editing. PB: Writing – review & editing. AH: Writing – review & editing.
